# Biodex Fall Risk Assessment in the Elderly With Ataxia: A New Age-Dependent Derived Index in Rehabilitation: An Observational Study

**DOI:** 10.1097/MD.0000000000002977

**Published:** 2016-03-11

**Authors:** Paola Prometti, Adriana Olivares, Giuseppina Gaia, Giampietro Bonometti, Laura Comini, Simonetta Scalvini

**Affiliations:** From the Unit of Recovery and Functional Rehabilitation (PP, GB); Laboratory of Cardiovascular Pathophysiology (AO); Laboratory of Clinical Biochemistry (GG); Health Directorate (LC); and Cardiac Rehabilitation Division and Telemedicine Service (SS), Scientific Institute of Lumezzane, Fondazione Salvatore Maugeri, IRCCS, Lumezzane, Brescia, Italy.

## Abstract

The aim of this study was to evaluate if the Biodex Fall Risk Assessment could provide an age-adjusted index useful for classifying patients at “risk of fall.”

This was a **c**ohort study conducted on 61 chronic patients, in stable conditions, having a history of ataxia, difficulty in walking or loss of balance, and aged >64 years. These patients were coming from home to our Institute undergoing a period of in-hospital standard rehabilitation. Assessment of clinical parameters was performed at entry. Functional scales (Functional Independence Measure [FIM] for motor and cognitive function, Barthel G, Tinetti POMA), and the Biodex Fall Risk Index (FRI) were performed at entry and discharge. The Normalized FRI, obtained adjusting FRI to the reported maximum predictive FRI for the relevant age, identified 2 types of patients: those with a greater risk of fall than expected for that age, labeled Case 1 (Normalized FRI>1); and those with an equal or even lesser risk of fall than expected for that age, labeled Case 0 (Normalized FRI≤1).

FRI, Normalized FRI as well as independent variables as age, sex, pathology group, FIM, BarthelG, were considered in a multiple regression analysis to predict the functional improvement (i.e., delta Tinetti Total score) after rehabilitation.

Normalized FRI is useful in assessing patients at risk of falls both before and after rehabilitation. At admission, the Normalized FRI evidenced high fall risk in 46% of patients (Case 1) which decreased to 12% after rehabilitation, being greater than age-predicted in 7 patients (Case 1–1) despite the functional improvement observed after the rehabilitation treatment. Normalized FRI evidenced Case 1–1 patients as neurological, “very old” (86% in age-group 75–84 years), and with serious events at 18 to 24 months’ follow-up. Normalized FRI, but not FRI, at admission was a predictor of improvement in Tinetti Total scores.

The normalized FRI effectively indicated patients at higher risk of fall, in whom health deterioration, falls, or cognitive decline was later documented at follow-up. The normalized FRI could be a standardized measure for identifying frailer patients becoming a further criterium of discharge home and marker of fall risk at home.

## INTRODUCTION

The risk of falling and sustaining injury due to a fall increases with age.^1,2^ Falls are not only associated with morbidity and mortality in elderly patients, but up to 60% of falls result in injury and have lasting effects that can lead to a subsequent restriction of basic activities of daily living (BADLs)^3^ and early admission to a long-term care facility.^4^ Falls and injury can induce a spiral of inactivity and decline that take older people below the critical “threshold” of performance in everyday activities. Risk factors that predispose the elderly to falling have now been identified. For community dwellers these include impaired mental status, use of psycho-active and multiple medications,^5^ visual impairment, lower extremity weakness, balance/gait impairments, and difficulties with BADLs.^3,6^

Intrinsic risk factors for falls, that is muscle weakness, poor balance, gait and functional ability, and fear of falling, are more common among the older age-group (over 80 years) and modifiable with tailored exercise,^7^ whereas extrinsic factors, that is social and physical factors related to the external environment, are more common in people under 75 years.^8^ While it is acknowledged that some risk factors are not modifiable (age, sex, social class, chronic medical conditions, irreversible vision problems), others such as physical activity,^3,9^ environment, and medication effects can be positively influenced through appropriate education and intervention.^10^

Regular physical activity (PA) is recognized as central for the prevention of several chronic diseases as well as to ameliorate the age-related decline in physical function. Both aerobic activity and muscular-skeletal exercise have been identified as important factors for the maintenance of good heal.^11^ There is now extensive evidence demonstrating that many falls are preventable with appropriate exercise. Most systematic reviews agree that a tailored program involving muscle strengthening, balance exercises, and a walking plan,^12^ prescribed by trained health professionals,^13^ is clearly effective.

In 2010, international guidelines on falls detailed specific programs for prevention of falls in the elderly.^10^ Research has shown that programs that include exercises that specifically challenge balance are more effective in preventing falls.^14–16^ The multidisciplinary program^6^ should also include interventions to mitigate fall risk factors such as medication to reduce postural hypotension, and Vitamin D (800 UI/die) supplements for all adults at risk.^17^

The Biodex Balance System (BBS) has been developed to evaluate and train the individual's capacity to maintain dynamic postural stability when subjected to a dynamic stress and the Fall Risk Index (FRI) is one of the BBS outputs.^18^ We aimed to evaluate if Normalized FRI, the age-adjusted index from FRI, constituting a novel approach for interpreting data, was able to better classify patients identifying those frailer before and after a standard rehabilitative program. The predictive role of Normalized FRI “at admission” on functional improvement was also evaluated.

## METHODS

### Participants

**FIGURE 1:**
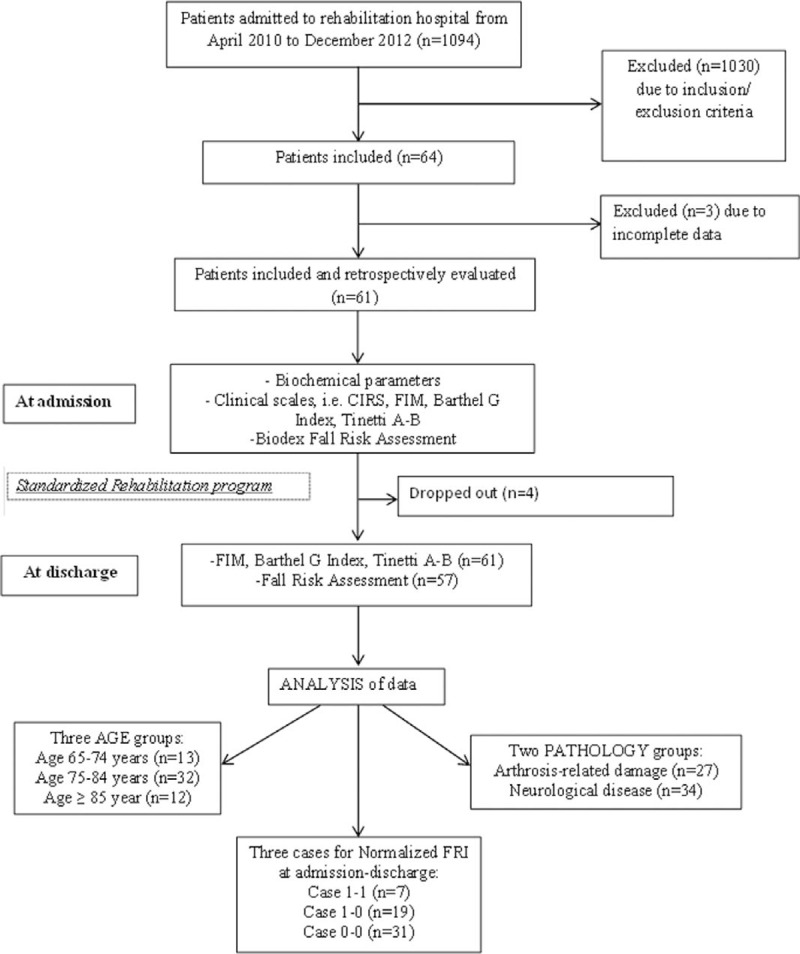
Design and flow of participants through the study.

This was a cohort study considering all patients, coming from home, consecutively admitted to the Rehabilitation Centre of Lumezzane, Brescia, Italy (part of the Salvatore Maugeri Foundation, FSM) for a usual rehabilitation program, between April 2010 and December 2012. A flow chart is shown in Figure 1.

Chronic patients in stable conditions having a history of ataxia, difficulty in walking or loss of balance, and aged >64 years were included in the study.

All patients with acute cerebrovascular disease were excluded. Postorthopedic surgery, postfractures patients or with cognitive decline were also not considered.

Data were retrospectively retrieved from the FSM archives and gathered into a single database for the analysis. For the current study, only the patients who had all the previous clinical inclusion criteria and medical data available from the Biodex Assessment evaluation were evaluated.

The study was conducted in accordance with the principles of the Declaration Helsinki. All patients, as part of our hospital admission procedure, gave informed written consent a priori for the use of their data for research.

### Intervention

All patients commenced the standard-care rehabilitation program (RP) the day after admission. It lasted about 4 weeks and involved a multidisciplinary team composed of physicians (physiatrists, geriatrists, internists), physiotherapists, and occupational therapist who decided the RP content on a patient-by-patient basis. Individually tailored rehabilitation activities, needs, specific goals set, and outcomes achieved were discussed at the time of the patient's admission and bimonthly by the team. The RP consisted of 330 min/week of motor exercises (6 days/week) and, in the last 2 weeks of the hospital stay, 150 min/week of occupational therapy (5 days/week). The motor RP was based on hip and knee range of motion (ROM), strengthening and conditioning exercises, bed-to-chair mobility, wheelchair skills, pre-gait (sit-to-stand, standing balance) and gait (parallel bars, walker, crutches) activities, bathroom skills, and ADL training. Physical therapy as well as ultrasound, laser, magnetic therapy, or transcutaneous electrical nerve stimulation (TENS) and electrical stimulation (Kotz) was individually tailored.

### Outcome Measures

#### Biochemical Examinations

Fasting blood samples were collected from all participants the day after admission. The local biochemical laboratory (plus an external reference laboratory for Vitamin D) performed the analyses: electrolytes such as calcium, magnesium, phosphorus, and albumin were assessed with colorimetric methods on an automated platform (COBAS INTEGRA, Roche diagnostics GmbH, Mannheim, Germany), while serum 25-OH Vitamin D was analyzed by Automated Analyzer (direct competitive method based on chemiluminescence—LIAISON 25 OH Vitamin D total, DiaSorin S.p.a., Saluggia, Vercelli, Italy).

#### Clinical Evaluations

Patients were administered the Cumulative Illness Rating Scale (CIRS) at admission. Other clinical scales of demonstrated reliability were administered at admission and discharge: Functional Independence Measure (FIM), Tinetti Performance-Oriented Mobility Assessment (Tinetti POMA), the Geriatric Barthel Index (Barthel G). In addition, the Biodex Fall Risk Index (FRI) was measured at admission and discharge.

1. The *Cumulative Illness Rating Scale* (CIRS)^19^ is used to evaluate comorbidities. This instrument measures disease burden in individuals with various chronic diseases. The CIRS-G provides a comprehensive review of medical problems concerning 14 organ systems, rating each organ system from 0 to 4. The instrument gives information about severity and comorbidity of chronic diseases. The present study considered the gravity index defined by the ratio between total score and either the number of different organ systems involved (CIRS 1) or the number of organ systems with score >3 (CIRS 2).
2. The *Functional Independence Measure* (FIM) assesses the patient's degree of independence and need of assistance in performing basic ADLs.^20^ It is an 18-item ordinal scale with 7 levels ranging from 1 (total dependence) to 7 (total independence). The FIM can be subdivided into a 13-item motor subscale and a 5-item cognitive subscale. The motor subscale score ranges from 13 to 91 (motor-FIM) and the cognitive score from 5 to 35 (cognitive-FIM). The maximum total score is 126 and refers to the best performance.
3. The *Tinetti Performance-Oriented Mobility Assessment* (Tinetti POMA)^21^ assesses patients’ balance from a clinical point of view. The POMA has a balance component (Tinetti A) and a gait (Tinetti B) component. Each item in the POMA scale is scored on a 3-point ordinal scale ranging from 0 to 2. The maximum balance score is 16, and the maximum gait score is 12 (high scores indicate best performance).
4. The Geriatric Barthel Index is used, like the FIM Scale, to assess the patient's degree of independence and need of assistance in performing basic activities of daily living (ADLs). We adopted the version of this scale with 0 to 20 score expressed as percentage.^22^ This index represents 10 items regarding ADLs (i.e., the ability to feed, to dress, to manage personal hygiene, etc.) and mobility (ability to move from the chair to the bed, to walk on level ground, to climb, and to go down stairs). The maximum total score is 20 and it refers to a situation of complete independence without any disability (0%).
5. The *Fall Risk Assessment* of the BBS (Biodex Medical Systems Inc, Shirley, NY) was performed to measure the dynamic balance index according to the manufacturer's instructions.^18^ The BBS was designed to evaluate and train neuromuscular control by assessing an individual's ability to maintain dynamic postural stability when subjected to a dynamic stress. It is a circular platform that moves freely and simultaneously about the anteroposterior and mediolateral axes. BBS induces joint stress providing a stimulus for a muscular response allowing the maximal stimulation of the mechanoreceptors of the ankle joint.^23^ Mechanoreceptors provide information on various environmental and physiological conditions that affect the person's ability to maintain equilibrium and prevent falls.^24^ Patients were instructed to maintain the vertical projection with their center of gravity in the midpoint of the platform by observing a vertical screen located 30 cm in front of their face. Each assessment took 20 seconds, with 10-seconds rest periods in-between. Patients stood barefoot on the platform with eyes open and the BBS was set to constant instability (Level 8). The average of the results from 3 assessments was obtained. The index of overall stability is measured in degrees (where 0° is the best possible value) and higher scores indicate poor dynamic balance. The evaluation was performed before and after the rehabilitation training. The results were expressed both as FRI (an absolute score that quantifies the risk of fall) and normalized FRI, a score obtained as the ratio between FRI and the maximum predictive FRI for the relevant age.^18^ The FRI age-adjusted ranges were provided by the BBS software and evaluated in a healthy and active people at different age. The normalized FRI identified 2 types of patients: those with a greater risk of fall than expected for that age, labeled Case 1 (Normalized FRI>1); and those with an equal or even lesser risk of fall than expected for that age, labeled Case 0 (Normalized FRI≤1).

### Statistical Analysis

All the present analyses have been carried out with the open source software R (R Core Team 2015).^25^ Baseline parameters were analyzed using descriptive statistics and their distributions were examined using the Shapiro–Wilk normality test. When distribution was parametric, data were presented as mean (standard deviation), otherwise as median value and interquartile range. Comparisons within group were performed using Wilcoxon signed rank test and between groups using Mann–Whitney test.

Analysis of subgroups (e.g., different pathologies and age groups) was performed by *χ*^2^ test with Monte Carlo correction (10^6^ iterations) for spared contingency tables.

Multiple regression with backward stepwise analysis was applied to evaluate a possible predictor of functional improvement (i.e., delta Tinetti total score) after rehabilitation program. The independent variables “at admission” were: age, sex, pathology group, FRI, Normalized FRI, Motor-FIM, Cognitive-FIM, and Barthel G Index. Analysis of standardized residuals was also carried out with scatchard plot, histogram, normal Q–Q plot, and Shapiro–Wilk normality test.

The significance level was set at *P* <0.05.

## RESULTS

Of 1094 patients admitted in the period to the Rehabilitation Institute, less than 30% (318) of them were from home. Only 64 fulfilled the selection criteria while 3 were excluded due to incomplete data (Figure 1). Sixty-one patients (mean age 79 ± 14 years, range 65–92; 74% women) constituted the study population. Baseline demographic and clinical characteristics are presented in Table 1. Patients had few comorbidities and moderate disability with lack of equilibrium associated with a depleted mineral metabolism (25-OH vitamin D, mean value 10.8 ng/mL, reference value for deficiency <20 ng/mL). Albumin value was low (normal range 3.5–5.2 g/dL), while calcium, magnesium, and phosphorus inside the normal range (calcium for age >61 years: 8.8–10.2 mg/dL, magnesium: 1.70–2.55 mg/dL, phosphorus: 2.7–4.5 mg/dL). Mean hospital length of stay (LOS) was 28 ± 6.8 days. The 2 main underlying pathologies, neurological and arthrosis-related disease, were equally represented in the overall study group and without significant differences in FRI and clinical scales except for cognitive FIM (*P* <0.001).

**TABLE 1:**
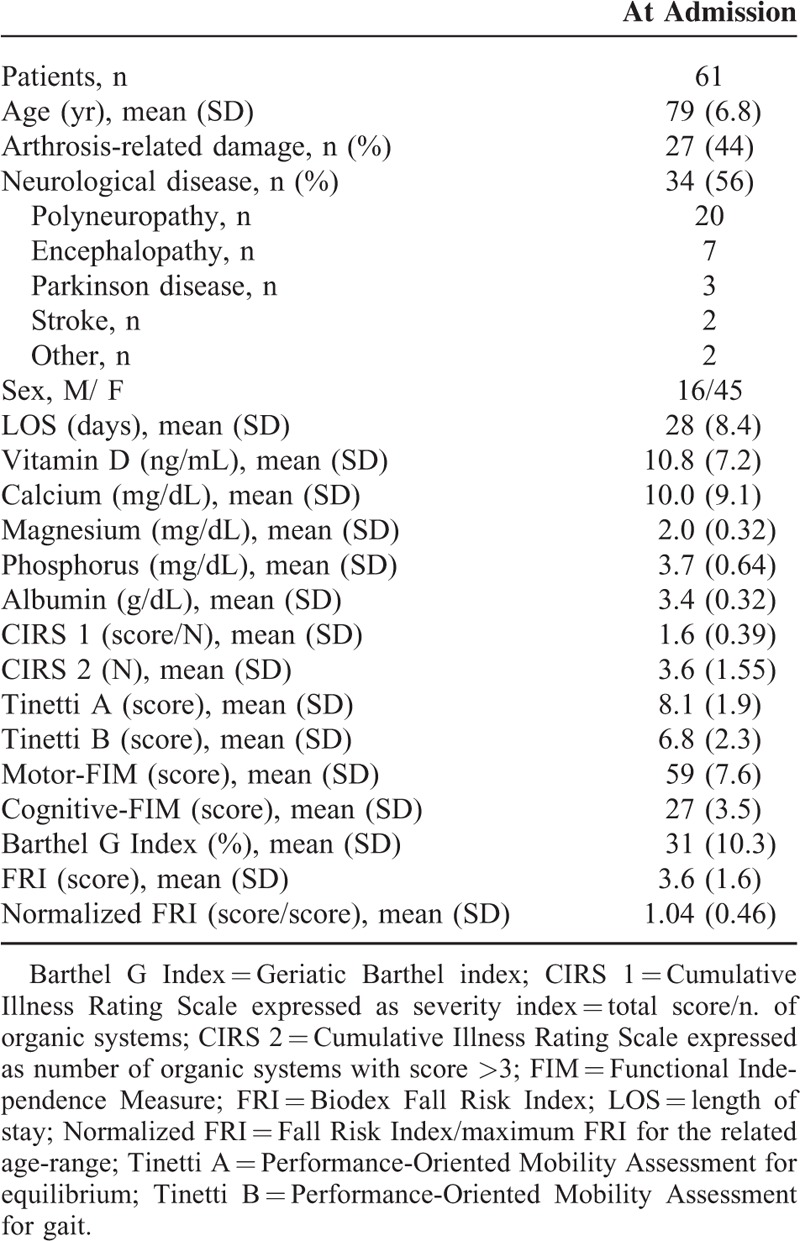
Patients’ Baseline Demographic and Clinical Characteristics

Table 2 shows the effect of the rehabilitation program on motor function, disability, and fall risk assessment in the overall study group. A significant improvement in functional performances was observed at discharge from rehabilitation in all parameters considered (for all, *P* <0.001). A significant improvement in FRI and normalized FRI (Table 2) was also seen in 57 out of the 61 patients who completed the balance assessment (both, *P* <0.001).

**TABLE 2:**
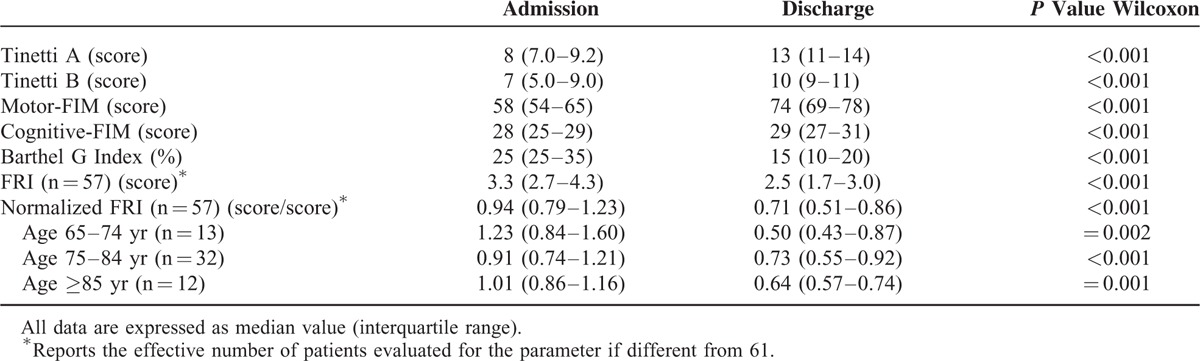
Comparison of Clinical Scales, Fall Risk Index, and Normalized Fall Risk Index (Stratified by Age) Between Admission and Discharge in the Whole Population

All patients were taking at least 1 medication associated with fall risk (benzodiazepines, antidepressant, antihypertensive or diuretic, medication associated with fall risk). Four patients experienced a fall during their stay: 1 of 7 (14%) was a Case 1-1, 1 of 19 (5%) was a Case 1–0, and 2 of 31 (6%) were Case 0–0.

**FIGURE 2:**
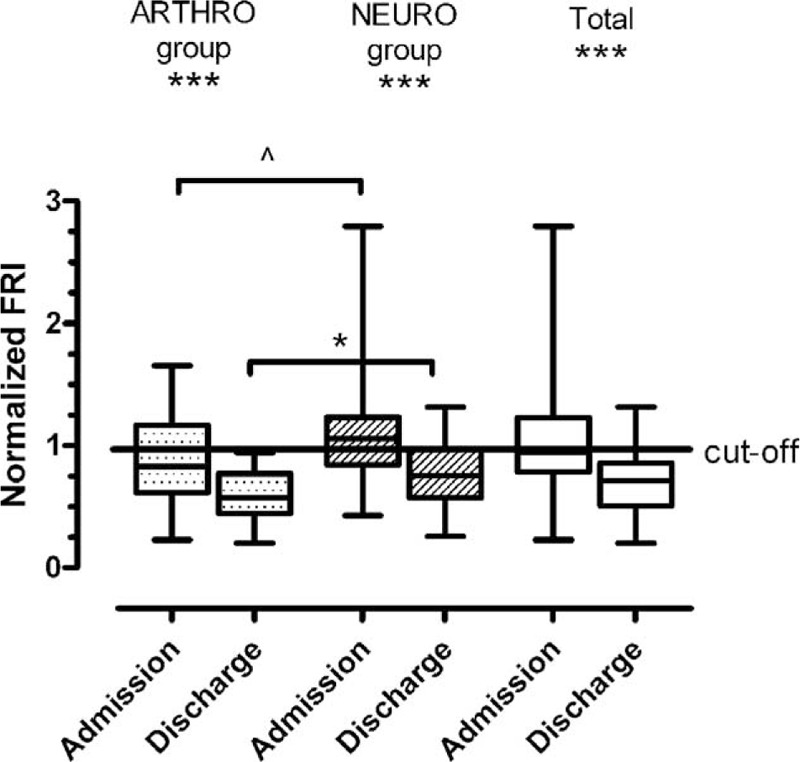
Comparison between pathology groups for normalized Fall Risk Index.^∗∗∗^*P* ≤ 0.001, ^∗^*P* = 0.04, ^^^*P* = 0.06.

The improvement after rehabilitation treatment was more deeply investigated through the analysis of the normalized FRI in patients’ different age groups and underlying disease. At admission, although not statistically significant, the normalized FRI was more impaired (Table 2) in the 65 to 74 years age-group and did not differ between the disease groups (Figure 2, *P* = ns**)**. At the end of rehabilitation, the normalized FRI improved independently from age (Table 2, for all, *P* <0.01) and disease (Figure 2, for all, *P* <0.001) with a statistically significant difference between the arthrosis-related and neurological groups (Figure 2).

The multivariate regression analysis indicated that, at admission, the normalized FRI, but not FRI, together with cognitive and motor FIM, predicted improvement in Tinetti Total score after the rehabilitative program. The regression model predicted 30% of the observed changes in Tinetti Total score (Table 3, for all, *P* <0.015). The standardized residuals resulted normally distributed (*P* = 0.47 at Shapiro–Wilk normality test).

**TABLE 3:**
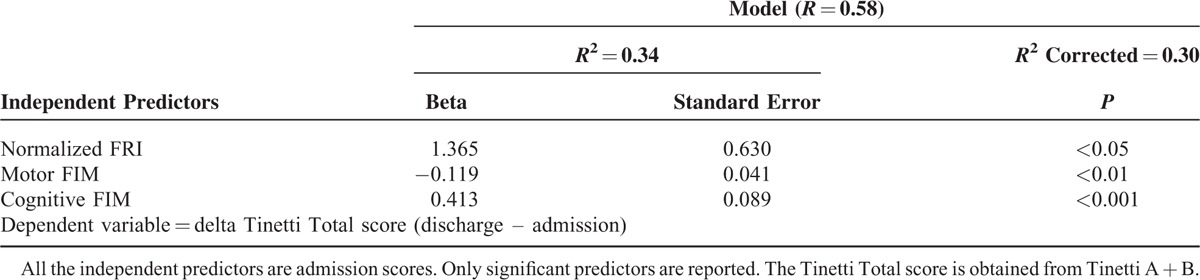
Predictive Model Through a Multivariate Linear Regression of the Delta Tinetti Total Score After a Usual Rehabilitative Program in Our Study Population

At entry, the normalized FRI identified 54% of patients as being in Case 0 group (no risk of fall) and 46% in Case 1 (risk of fall). At the end of the rehabilitation, the majority of patients (88%) were Case 0, while Case 1 patients decreased to 12%. Considering Cases 0 and 1 with respect to admission/discharge the possible clinical scenarios were four:

1. Cases 0–1: unstable patients with a worsened fall risk after the rehabilitation treatment.
2. Cases 0–0: stable patients with a fall risk profile as age-predicted before and after the rehabilitation treatment.
3. Cases 1–0: improved patients with a reduction of fall risk to normalization after the rehabilitation treatment.
4. Cases 1–1: unchanged patients with a fall risk greater than age-predicted despite the rehabilitation treatment.

There were no Case 0–1 patients. For Cases distribution according to pathology there was a significant difference evaluated by *χ*^2^ corrected (*P* = 0.04) between groups. Case 1–1 patients constituted 8% of the 65 to 74 years age-group and 19% of the 75 to 84 years age-group and were all neurological patients.

Table 4 shows clinical scales, FRI and normalized FRI at both admission and discharge in the 3 different cases. Functional and disability parameters significantly improved after rehabilitation in all Cases considered (for all, *P* < 0.05). On the contrary, FRI and normalized FRI significantly improved only in Case 0–0 and Case 1–0 (both, *P* <0.001).

**TABLE 4:**
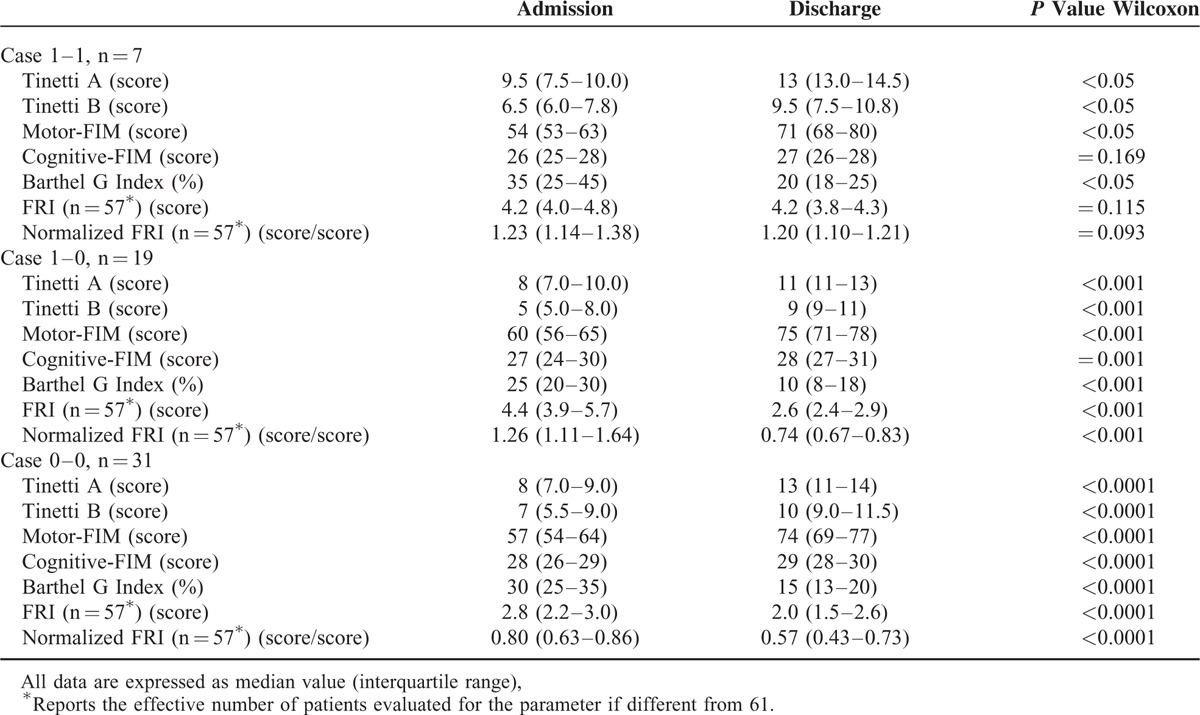
Comparison of Clinical Scales, Fall Risk Index, and Normalized Fall Risk Index Between Admission and Discharge in the 3 Different Cases

**FIGURE 3:**
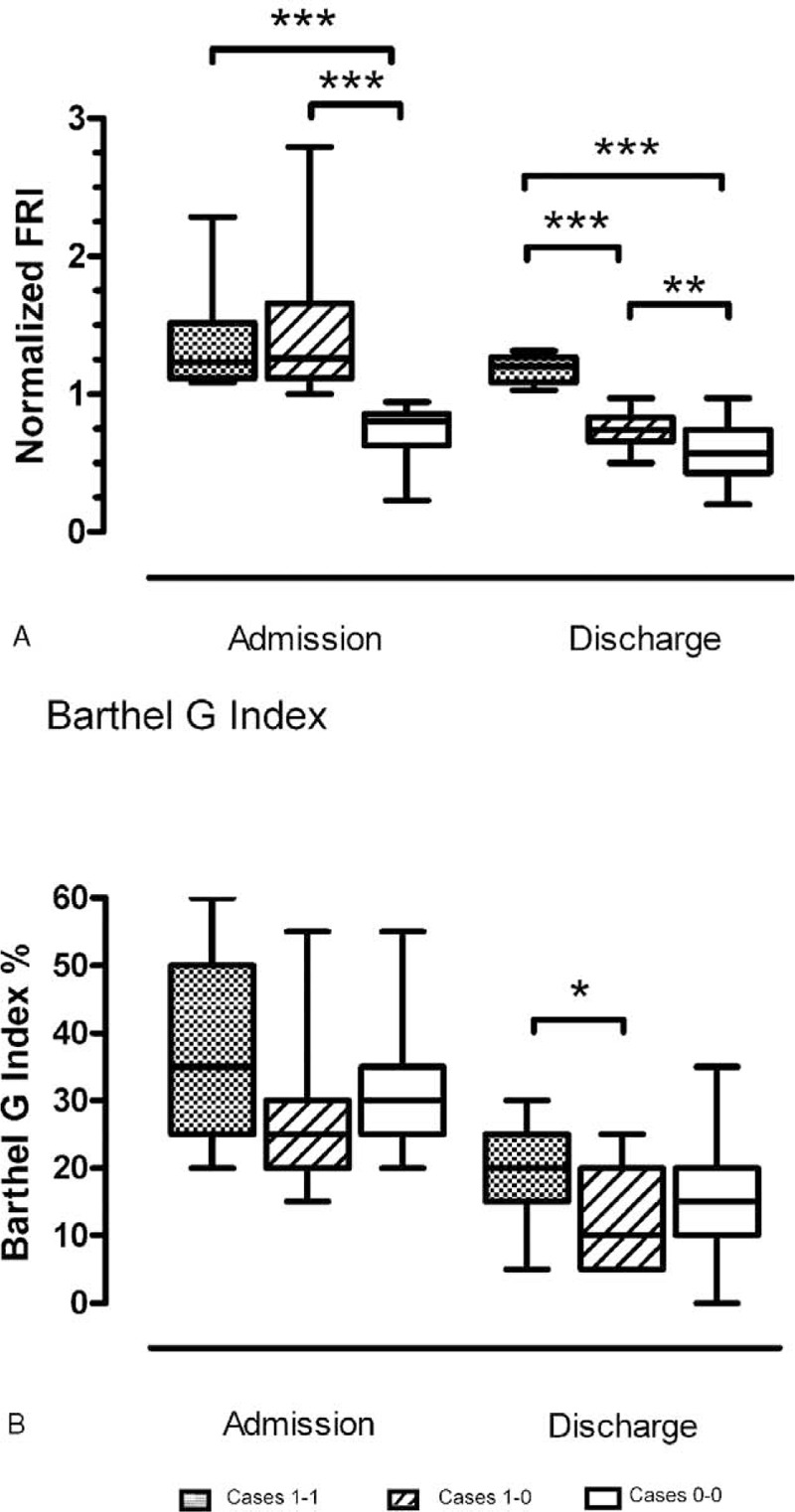
Comparison between cases at admission and discharge for 2 relevant parameters. ^∗∗∗^*P* ≤ 0.001, ^∗∗^*P* = 0.003, ^∗^*P* = 0.03.

Independently of age and underlying disease, Case 1–1 patients (n = 7) were compared with Case 1–0 (n = 19) and Case 0–0 patients (n = 31) for all clinical scales or indexes. Only the normalized FRI and Barthel Index differed significantly between the Case groups (Figure 3). In the light of these results, we retrospectively reevaluated at a distance of 18 to 24 months from hospital discharge all Cases defined as 1 at admission (i.e., both the 7 Cases 1–1 and the 19 Cases 1–0). Concerning the 7 Case 1–1 patients, 3 had had a clinically important ischemic ictus, 1 a rapid cognitive decline with bed rest, 2 had experienced repeated falls at home and 1 patient, even though stable from the neurologic and motor point of view, had undergone invasive procedures of myocardial revascularization. In contrast, the 19 Case 1–0 patients at 18 to 24 months showed a situation of clinical stability with no critical health events reported in any patient over the period (Table 5).

**TABLE 5:**
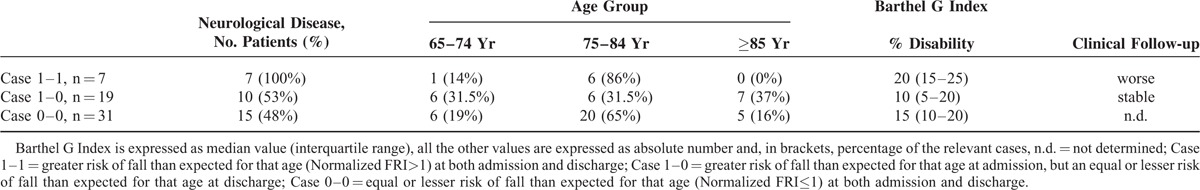
Comparison of the 3 Cases of Normalized FRI (at admission Vs. discharge) With Respect to Underlying Pathology (Proportion of Patients With Neurological Disease), Age Group, Barthel G Index at Discharge and Clinical Follow-Up

## DISCUSSION

This study shows for the first time that the Fall Risk Index adjusted for age can identify, among elderly patients, those (identified as Case 1–1) who, although they have recovered their motor function at the end of rehabilitation, are frailer and at higher risk of falls and for this reason need more attention for discharge criteria and fall risk at home.

The elderly population admitted for rehabilitation to our Institute presented moderate disability and a deficit in vitamin D in line with previous studies showing that it is often underestimated in elderly people.^17^ For this reason our patients were given vitamin D supplementation during the rehabilitation. No further evaluation on vitamin D was carried out at discharge.

Functional tests evaluated after rehabilitation, in line with other studies, evidenced a global improvement in all patients irrespective of their underlying disease^26,27^ or age.^28^

In this study, the focus was particularly pointed at balance control with the use of the BBS. While the rehabilitation program that patients underwent was the usual one provided in the Institute, assessment of balance using the BBS constituted a novelty for the patients’ evaluation concerning risk of falls. Different papers have dealt with the need for assessment of risk of falls,^28^ but only a few refer to Biodex.^29–31^ Although there is a large recognized need for consensus, measurement indexes for balance/risk of fall are not standardized as yet. Moreover, no commercially available posturography device emerges as gold standard of balance assessment,^32^ useful for comparison. We focused our attention on the FRI, one of the output parameters of the BBS tests of equilibrium stability, and found an improvement in all patients with different age and disease, similar to results of previous studies using BBS.^29,31^

It was decided to investigate if FRI index improvement could be enough to describe patients “not at risk of fall.” To answer this question, we introduced the age-adjusted FRI. Using it the extent of the clinical improvement in FRI was better characterized, allowing identifying frailer patients at admission (Cases 1) and patients who remained frailer despite the rehabilitation treatment (Cases 1–1). At discharge, functional scales, FRI and Normalized FRI improved for all age- and disease groups. The number of patients who were considered Case 1 at admission declined as well. However, few remaining Cases 1–1 were associated with deterioration when evaluated at 18–24 months retrospective follow-up.

In addition, Normalized FRI at admission resulted as a predictor of improvement, quantified in a positive delta Tinetti Total (discharge score – admission score), being total score of Tinetti a reliable and valid tool for assessing balance and gait statum and fall risk in individuals.^33,34^ In the study, a substantial amount of positive change in Tinetti Total could be predicted by the initial dynamic balance and cognitive status of patient. Poor dynamic balance in Normalized FRI (high scores), and poor scores in Motor-FIM (low scores) associated with better Cognitive FIM (high scores) predict improvement in Tinetti Total. That is, patients in worse balance and preserved cognitive condition at admission are the ones obtaining the more benefit from rehabilitation.

The Barthel G Index at discharge was different and better for Cases 1–0 with respect to Cases 1–1. This may be due to the fact that Barthel G, quantifying disability in daily life activities, is strongly conditioned by the pathology where the disability is greater^35^ and Cases 1–1 were all neurological patients. These cases could have obtained a better recovery prolonging the rehabilitation period (LOS of the present study was 28 days) through an intermediate evaluation of the normalized FRI.

Despite the above-mentioned differences among Cases, looking at the overall study population, an incontrovertible improvement of FRI after treatment in the very old patients was observed. In patients >85 years, the FRI improvement matched to age was surely high and allowed the normalization of FRI in more than 58% of patients. This suggests that rehabilitation treatment was particularly useful for these patients,^28^ who are often less considered than younger patients because of their frailty, major risk of injury, or complications during physical exercise and care costs.

The Biodex Balance System has a wide range of clinical applications. In the present study, this tool was approached first to provide a more objective measure for following up patients during rehabilitation. Second, as part of a comprehensive Fall Risk Assessment Program,^26^ the clinical application of the normalized FRI could establish a more appropriate basis for discharge criteria and fall risk at home. Third, application of Biodex Balance in rehabilitation could be an additional tool for providing exercises to improve the standard rehabilitation program for various pathologies and particular patients’ needs.

## LIMITATION OF THE STUDY

The absence of a control group has limited the interpretation of the measured improvement after rehabilitation: only a comparison between improvement at different age and pathology BBS evaluations were performed in a reduced number of patients with respect to those admitted to the Rehabilitation Centre, because respecting the Regional rules, more than 70% of patients admitted to our Institute were from hospital. Moreover, within the included patients only 20% of them performed the BBS assessment due to a reduced availability of BBS trained operators. The small sample size of Cases 1–1 could have biased interpretation of the results. In addition, data collected from a single-center study could limit the generalizability of our findings. The use of FRI normalization is a novel approach for interpreting data. This constitutes strength of our study but, by the same token, the possibility of comparison with other studies is currently ruled out.

## CONCLUSIONS

Performing an initial assessment of balance control is crucial for addressing balance deficits associated with aging and clinical diagnosis. In the present study, the normalized FRI results to be helpful in testing the risk of falls in patients at admission to a program of in-hospital rehabilitation in addition to other usual tests. Moreover, the normalized FRI could be a standardized measure for identifying frailer patients at higher risk of falls, becoming a further criterium of discharge home (i.e., in-hospital prolonged length of stay versus return home). Further studies are warranted to better explore this field.
